# The Impact of Recent Collective Trauma in Israel on Eating Disorders and Disordered Eating: An Overview and Clinical Perspective

**DOI:** 10.3390/healthcare14131908

**Published:** 2026-07-01

**Authors:** Yael Latzer, Daniel Stein

**Affiliations:** 1 School of Social Work, Faculty of Social Welfare and Health Sciences, University of Haifa, Haifa 3498838, Israel; 2 Eating Disorders Institution, Psychiatry Division, Rambam Health Care Campus, Haifa 3109601, Israel; 3 Pediatric Psychosomatic Department, Safra Children’s Hospital, Sheba Medical Center, Tel Hashomer, Ramat Gan 5262100, Israel; prof.daniel.stein@gmail.com; 4 Maayanei Hayeshua Medical Center, Bnei-Brak 5154475, Israel; 5 Department of Psychiatry, Gray Faculty of Medical and Health Sciences, Tel Aviv University, Tel Aviv 6997801, Israel

**Keywords:** eating disorders, trauma and stressor-related disorders, collective trauma, COVID-19, October 2023 war, telemedicine, health services, Israel

## Abstract

**Background:** Periods of collective crises are associated with substantial deterioration in mental health. Whereas trauma-related psychopathology has received considerable clinical and policy attention during such periods, eating disorders (EDs) and disordered eating (DE) remain comparatively under-recognized. EDs and DE are well recognized in the Israeli context; however, accumulating evidence indicates a significant increase in their prevalence during periods of collective crisis. **Objective:** We sought to synthesize empirical findings and clinical observations in Israel about the impact of the COVID-19 pandemic and the October 2023 War on the emergence and exacerbation of DE and EDs, and to examine the implications for clinical practice and mental health policy. **Methods:** We conducted a narrative review integrating epidemiological data, findings from Israeli healthcare organizations, scientific studies, and clinical insights from specialized ED services. **Results:** Across both crises, Israel experienced a marked increase in the full spectrum of eating-related disturbances, ranging from emotional eating and subclinical DE to severe full-blown EDs. Adolescents and young adults were particularly affected, but increased ED-related disturbance was also observed among adults and individuals with pre-existing EDs. These findings coincide with a prolonged duration period before receiving treatment and with limited service capacity. In response, the mental health system attempted to implement rapid adaptations, including expanded telemedicine, short-term targeted interventions, and new ambulatory and day treatment programs. **Conclusions:** Our findings indicate that DE and EDs constitute a significant and escalating mental health burden during periods of collective crises that is comparable to other trauma-related conditions. Just as comprehensive short- and long-term intervention frameworks have been developed for trauma-related conditions, similar models are urgently required for EDs.

## 1. Introduction

Israel is a society in transition, shaped by ongoing demographic, cultural, and socio-political changes. This state of transition has intensified, particularly in the context of two major collective traumatic crises, including the COVID-19 pandemic (2020–2022) and the unprecedented terrorist attack of 7 October 2023, followed by a prolonged multi-front war. Both crises imposed an extraordinary burden on Israel’s already strained medical and mental health systems.

Collective crises may influence eating-related disturbances through interconnected pathways involving trauma exposure, psychological distress, and coping processes. In this context, maladaptive eating behaviors may serve as attempts at emotional regulation during prolonged stress and uncertainty.

Exposure to collective trauma may lead to heightened psychological distress, including anxiety and posttraumatic stress symptoms, alongside disruption of daily routines and social support systems. In this context, maladaptive eating behaviors may emerge as coping strategies aimed at regulating distress, restoring a sense of control, or managing uncertainty during stressful periods. These processes may contribute to both the exacerbation of pre-existing eating disorders (EDs) and the emergence of new eating-related disturbances.

The COVID-19 pandemic was characterized by widespread fear of an unfamiliar and potentially lethal illness, accompanied by heightened psychological distress, fear of contagion, anxiety, depressive symptoms, and insomnia. Prolonged lockdowns and social distancing measures further intensified social isolation [1,2], leading to a marked increase in diverse psychiatric pathologies [1,3], and increased rates of suicidal ideation and suicide attempts [2,4]. However, findings regarding death by suicide during the pandemic were mixed, with some studies reporting an increased risk [1] and others showing no significant changes [4].

These findings are consistent with international reports indicating increased ED symptomatology during the COVID-19 pandemic [5,6].

Recent surveys of adult patients with anorexia nervosa (AN) and bulimia nervosa (BN) during the COVID-19 pandemic showed greater reported frequency of food restriction, binge-eating episodes, and purging behaviors, as well as an increase in eating, shape and weight concerns, and a drive for physical activity. Alongside these symptoms, patients reported heightened overall anxiety and depression and a reduction in well-being, likely related to economic difficulties, problems with family, and overall social isolation [7,8,9,10,11].

War conditions are also associated with heightened psychological distress among civilian populations and increased rates of psychiatric morbidity compared to pre-war periods [12,13]. However, unlike the profound isolation that characterizes pandemics, wartime contexts may foster greater social cohesion and collective solidarity, particularly when war is perceived as justified or necessary. Historically, such circumstances have been linked to lower rates of suicidal behavior compared to pre-war periods [14].

On 7 October 2023, Israel experienced a large-scale and unprecedented terrorist attack perpetrated by Hamas, resulting in the murder of approximately 1200 people, most of whom were civilians, and the abduction of 240 soldiers and civilians into Gaza [15]. Subsequently, hostilities expanded to Israel’s northern regions with sustained fighting against Hezbollah, leading to approximately 3000 civilian casualties and 127 civilian deaths. During the October 2023 war, approximately 68,500 Israelis from 43 communities were evacuated from their homes. More recently, on 25 June 2025, a 12-day escalation involving Iran resulted in the destruction of over 1000 residential buildings, the evacuation of approximately 10,000 additional civilians, more than 1000 casualties, and the deaths of approximately 30 Israeli civilians [16]. On 28 February 2026, another war with Iran escalated, not terminating at the conclusion of this article.

The October 2023 war has differed markedly from previous Israeli wars, which were typically short and characterized by a broad public consensus on their necessity. In contrast, the current war is the longest in Israel’s history (approximately two and a half years and still ongoing) accompanied by profound societal polarization and ongoing debate regarding its duration, justification, and goals. The war has affected the civilian population more extensively than any previous conflict, exposing large segments of society to prolonged uncertainty, threats, and disruption.

Studies conducted during the first months following the 7 October attack documented a sharp and alarming rise in mental health morbidity across all population groups, with particularly severe effects observed in regions directly exposed to the hostilities [13]. This deterioration was driven not only by direct exposure to terror and ongoing military activity but also by widespread displacement, cumulative losses, and the prolonged anguish experienced by the families of individuals held captive in Gaza [16].

Recent findings further indicate that nearly one-third of the Israeli population met the criteria for probable post-traumatic stress disorder (PTSD) during the months following the attack [17]. Given the well-established association between PTSD symptoms and an elevated suicide risk [18], these findings are of particular concern. In a longitudinal study assessing 710 Israeli civilians two months prior to the war (August 2023) and one month after its onset (November 2023), Levi-Belz et al. [17] demonstrated a significant increase in suicidal ideation, closely linked to a parallel rise in post-traumatic symptoms.

It is important to understand that the present analysis is being conducted in close temporal proximity to the 7 October 2023 attack and the ongoing war that followed. At the time of writing, Israel continues to face ongoing military escalation, including sustained missile attacks from Iran and Hezbollah in Lebanon, affecting large parts of the country.

Israeli civilians continue to be exposed to prolonged conditions of threat, uncertainty, displacement, and disruption of daily life. Under these circumstances, the full psychiatric consequences of this collective trauma cannot yet be fully understood. The trajectory of trauma-related psychopathology, including PTSD symptoms and their interaction with disordered eating (DE) and EDs, will likely become clearer only through longitudinal observation. Accordingly, the current findings should be interpreted as early indicators of an evolving crisis rather than definitive estimates of long-term mental health outcomes. These findings are consistent with a growing body of international research that has demonstrated a robust association between trauma exposure, PTSD symptoms, and DE behaviors [19,20,21]. Against this background, the present study examines the influence of the COVID-19 pandemic and the October 2023 war on the development and exacerbation of DE and full-blown EDs.

Eating-related disturbances are commonly viewed as existing along a continuum, ranging from maladaptive dieting, emotional eating, and subclinical DE to full-threshold EDs. Emotional eating has been conceptualized as a maladaptive coping strategy in response to stress and negative effects [22,23]. This perspective highlights the importance of examining both DE and EDs to capture the full spectrum of eating-related difficulties. In the present review, the term eating disorders (EDs) refers to full-threshold clinical disorders meeting established diagnostic criteria. Disordered eating (DE) refers to subclinical maladaptive eating attitudes and behaviors that do not meet full diagnostic criteria. The broader term eating-related disturbances is used as an umbrella term encompassing EDs, DE, emotional eating, unhealthy dieting, and other maladaptive eating-related behaviors.

The COVID-19 pandemic and the ongoing war following 7 October crises appear to have amplified psychological vulnerability across diverse population groups, contributing to a notable increase in the full spectrum of eating-related disturbances, from emotional eating and subclinical DE to severe, full-blown EDs, while simultaneously catalyzing the emergence of novel therapeutic responses aimed at addressing the escalating clinical demand.

Although both collective crises involved elements of fear and existential threat, the current context in Israel is unique in its characteristics and its intensity. It includes continuous exposure to danger, life-threatening conditions, displacement, the loss of loved ones, and prolonged uncertainty. Therefore, although both events are important to consider, their distinct natures warrant separate yet complementary examinations to understand their impact on eating-related pathology.

The extent and intensity of these conditions are of particular relevance to mental health, as prolonged exposure to threats, uncertainty, and disruption has been consistently associated with increased psychological distress and vulnerability to maladaptive coping behaviors, including eating-related disturbances.

Given the rapidly evolving nature of the current war and the relative scarcity of systematically collected data, a narrative review approach was deemed most appropriate. This methodology allows for the integration of heterogeneous sources, including emerging empirical studies and clinical observations, and enables a timely and context-sensitive synthesis of evidence in a field where systematic data are limited.

This paper presents a narrative overview of the literature, focusing on empirical studies conducted in Israel during the COVID-19 pandemic and the ongoing October 2023 war, as well as clinical observations with respect to AN, BN, binge ED, and emotional eating.

The aim of the present paper is to provide an integrative overview of the impact of the COVID-19 pandemic and the ongoing war following 7 October on EDs, DE, and broader eating-related disturbances, including emotional eating, in Israel, while incorporating both empirical evidence and clinical perspectives. In addition, this paper seeks to highlight the implications for prevention, intervention, and mental health policy in the context of prolonged and cumulative stress.

## 2. Methods

This study provides a narrative overview of the literature. A structured search was performed in major academic databases, including PubMed, PsycINFO, and Google Scholar, to identify relevant studies published between 2020 and early 2026.

The search terms included combinations of keywords such as “eating disorders,” “disordered eating,” “COVID-19,” “war,” “trauma,” and “Israel.” Studies were included if they examined EDs or DE in the context of the COVID-19 pandemic or the ongoing war in Israel after 7 October. Both empirical studies and relevant clinical reports were considered for inclusion.

Given the emerging and heterogeneous nature of the literature, formal systematic review procedures (e.g., PRISMA guidelines) were not applied to this review. Instead, a narrative approach was chosen to integrate diverse sources, including early empirical findings and clinical observations of patients with eating disturbances.

In addition, informal consultations were conducted with Israeli ED specialists (approximately 15 clinicians) to obtain clinical insights into treatment adaptations and service delivery during crises. These consultations were not part of a formal qualitative study but were used to complement the interpretation of existing literature.

Although this study was not conducted as a formal systematic review, we applied a structured and transparent approach to identify and select the literature. Studies were screened based on their relevance to the aims of the review, with priority given to empirical studies conducted in Israeli populations and addressing EDs and DE in the context of collective crises. Where available, preference was given to studies with larger samples, longitudinal designs, or data derived from national healthcare systems. In addition, relevant clinical reports and expert-based publications were included to capture emerging trends in a rapidly evolving context where systematic data remains limited.

## 3. Results

### 3.1. Historical, Cultural, and Intergenerational Contexts of Eating and Trauma in Israeli Society

Israel represents a unique socio-cultural context characterized by the intersection of ancient traditions, rapid modernization, and ongoing exposure to collective stress and existential threat. As a multi-cultural society composed of diverse ethnic, religious, and immigrant groups, Israel provides a complex setting in which eating behaviors, body image, and eating-related disturbances are shaped not only by individual psychological factors but also by deeply embedded cultural and historical influences. These include longstanding collective traumas, most notably the Holocaust, repeated wars, and chronic exposure to terrorism, which continue to shape both individual and societal experiences of threat and vulnerability, but also of growth and survival.

Within this context, the concept of intergenerational transmission of trauma is particularly salient. Research suggests that exposure to historical and collective trauma may shape patterns of emotional regulation, perceptions of safety, and coping mechanisms across generations. In such settings, the body may become a central site for the expression and regulation of distress. Eating-related disturbances can therefore be understood not solely as psychopathological phenomena and at-risk behaviors [24] but also as culturally embedded responses to both acute and chronic stress, reflecting attempts to regain control, establish boundaries, or cope with overwhelming effects [25].

Moreover, Israel’s multi-cultural heterogeneity further complicates the relationship between trauma and eating behaviors. Distinct subgroups—including ultra-Orthodox Jewish communities; Muslim and Christian Arab Israeli populations; secular groups; and immigrant communities from many countries around the globe—differ substantially in their attitudes toward the body, food, and mental health, as well as in patterns of help-seeking behaviors and access to care [26]. For example, studies among ultra-Orthodox populations highlight the tension between adherence to strict religious and communal norms and increasing exposure to Western ideals of thinness and self-realization, a dynamic that may heighten vulnerability to DE in certain individuals. At the same time, deep inherent Jewish beliefs, strong communal structures, and religious frameworks may serve as protective factors, underscoring the complex bidirectional role of culture [27].

Within many Jewish cultural contexts, food occupies a central symbolic and relational role, extending beyond its nutritional function. Food is deeply intertwined with family life, rituals, memory, and collective identity, often serving as a primary medium for expressing care, continuity, and belonging [28]. In the aftermath of collective trauma, food may simultaneously function as a source of comfort and emotional regulation, as well as a domain in which fear, control, restriction, or dysregulation are enacted. This dual role of food, as both a regulator of distress and a potential site of pathology, adds an additional layer of complexity to the understanding of eating-related disturbances in the Israeli context [29].

Taken together, these cultural, historical, and intergenerational dimensions suggest that DE and EDs in times of crisis do not emerge in a vacuum. Rather, they are embedded within a multilayered socio-cultural landscape in which past and present traumas, cultural meanings of the body and food, and varying community norms and frameworks interact dynamically to either protect against or increase the risk for the development of DE or EDs. Recognizing this broader context is essential for developing culturally sensitive clinical approaches and for interpreting observed changes in eating-related psychopathology during periods of collective crisis, including the current traumatic conditions [30].

### 3.2. Eating-Related Disturbances in Israel During the COVID-19 Pandemic

During the COVID-19 pandemic (2020–2022), Israel faced an unprecedented health and social crisis marked by prolonged lockdowns, social distancing measures, and school and public institution closures. In the context of DE and EDs, these abrupt disruptions in daily life created significant and sustained stressors for children, adolescents, and young adults already struggling with pre-existing eating-related difficulties. The unique convergence during the pandemic of confinement, social isolation, heightened psychological distress, disruption of eating, sleeping, and physical activity routines, increased boredom, greater body- and appearance-related preoccupation, and intensified exposure to social media, created fertile ground for the exacerbation of pre-existing eating-related symptoms, contributing to an overall increase in the prevalence of DE, especially among adolescents and young adults [31].

Indeed, during the pandemic years, a sharp increase was observed in ED-related cases and referrals around Israel, particularly among young people, alongside a marked rise in hospitalizations for EDs, most notably for AN. These trends were accompanied in the referred people by greater medical instability, more severe ED symptomatology, and poorer overall psychological and behavioral adaptation. The country’s largest healthcare organization, Clalit Health Services, which insures approximately 70% of the population, reported a 20–100% increase in requests for ED-related treatment services during 2020 compared to 2019, resulting in a substantial prolongation of waiting times, in some cases extending to 4–12 months. This wide range reflects variability across service types, with more moderate increases in waiting time observed in outpatient services and substantially higher increases observed in acute and specialized treatment settings [32]. Similarly, data from another Israeli healthcare organization indicated that the number of adolescents seeking treatment for EDs in 2020 increased by 56% compared with 2019 [32].

Further evidence emerged from a large-scale study conducted at Maccabi Healthcare Services, the second-largest healthcare organization in Israel, which examined the annual rate of new outpatient ED visits among adolescents aged 12–17 years between 2017 and 2021. As shown in Table 1, a substantial increase in new ED-related visits was observed in 2020 and 2021 compared with the pre-pandemic period (2017–2019) among both girls and boys. These findings suggest that whereas the increase in DE and other eating-related disturbances among girls during the pandemic might have been linked to gender-specific vulnerabilities, such as heightened body image concerns associated with weight changes, reduced physical activity, and increased snacking during lockdowns, the parallel increase observed among boys might reflect the broader impact of the pandemic as a prolonged stressor that amplifies risk-taking and maladaptive coping behaviors, including maladaptive eating [24,33,34].

A similar pattern was evident for specialized clinical services. A substantial increase in ambulatory ED interventions was documented between 2019 and 2021 at Sheba Medical Center, Tel Hashomer, Israel’s largest tertiary medical center. As shown in Table 2, 2021 was characterized by a marked rise, relative to both 2019 and 2020, in the number of treatment sessions delivered, ambulatory patients treated, and new patient admissions to the center’s specialized ED outpatient services for both adolescents and adults. This increase occurred despite a modest decline in the number of patients in 2020 [31].

Hospital-based services were also significantly affected by the pandemic. One Israeli study reported that during the first year of the pandemic, hospitalizations in a pediatric department because of AN among adolescents increased by a factor of 2.07 compared to the annual average in preceding years (94 patients in the 1st 12 months of the COVID-19 pandemic vs. a yearly mean of 45.25 during 2015–2019 [35]). Notably, the physical severity of the illness at the time of admission was not higher than that in the pre-pandemic periods, this being attributed to enhanced parental supervision and earlier symptom detection during the lockdowns, when families spent more time together [35].

Beyond these quantitative increases, the pandemic profoundly impacted the psychological well-being of individuals with EDs. In a study assessing self-perceived substance and behavioral addictions among Israeli adolescents during the COVID-19 pandemic, 34% reported binge eating behaviors [34].

Another Israeli study conducted at the onset of the COVID-19 lockdown among 63 patients with various ED diagnoses found marked disturbances in eating behaviors, accompanied by moderate levels of depressive symptoms and relatively low levels of anxiety and stress [36]. The authors hypothesized that intense preoccupation with eating- and body-related concerns might have functioned as a psychological buffer, partially diverting attention away from external stressors associated with the lockdown. In contrast, a later study conducted between 2020 and 2021 demonstrated that both adolescent and adult women with AN reported significantly elevated levels of ED symptoms, anxiety, depression, and PTSD symptoms compared to non-ED control groups [31]. Participants also described heightened pandemic-related emotional and behavioral distress, such as health anxiety and distress related to social isolation, with greater pandemic-related distress being strongly associated with more severe ED symptomatology and lower psychological resilience [31]. Importantly, adult women with AN exhibited greater psychological and physical symptom severity and lower resilience than adolescents during the pandemic [31].

A longitudinal study [37] was conducted among 313 adults in Israel at three time points, beginning with the first COVID-19 lockdown (April 2020) and extending through June 2020. In each wave, participants were asked to report on exercise, fruit and vegetable intake, family meal sharing, and screen time. There was an initial increase in the frequency of exercise and shared meals, followed by a significant decrease in both behaviors. Engaging in more exercise and less screen time had a positive impact on participants’ psychological health. The authors concluded that external events, such as the COVID-19 lockdowns, may have influenced eating-related health behaviors, which, in turn, influenced psychological health. Moreover, higher engagement in healthy lifestyle behaviors (e.g., exercising and eating fruits and vegetables) prior to the COVID-19 pandemic was significantly correlated with higher adherence to coronavirus protective behaviors and better overall self-assessed health [38].

Healthy eating behavior during COVID-19 was compromised. An international survey conducted by 35 research organizations from Europe, Western Asia, North Africa, and America found that many respondents changed their eating patterns during the pandemic, with increased unhealthy food consumption, out-of-control eating, and snacking [39]. Such behavior was most probably a mood-driven consequence of increased boredom associated with the pandemic [40].

Along these lines, in a study comparing the eating behaviors of 453 Israeli and Maltese psychology and social work students during the COVID-19 pandemic [41], almost 70% of respondents in both groups reported eating more salt- and sugar-loaded foods due to COVID-19. Additionally, approximately 50% of the respondents reported weight gain. Moreover, due to COVID-19, most respondents (74.3%) reported a deterioration in their psycho-emotional well-being in the previous month; however, no significant differences were found across countries or religiosity. Furthermore, no significant differences were found in changes in eating behavior and weight increase across countries and religiosity status.

Across the two countries, respondents who reported eating more salt- and sugar-laden food because of COVID-19 had significantly higher fear of infection and burnout and lower resilience. In addition, respondents who experienced changes in eating behavior during COVID-19 reported more previous-month substance use and deterioration of psycho-emotional well-being.

Two other Israeli studies [33,42] examined the relationship between several lifestyle changes (including changes in eating habits) and the presence of psychological distress (e.g., depression or anxiety) and emotional eating in an online survey of 1969 respondents during the COVID-19 pandemic. People with positive and negative COVID-19-related lifestyle changes demonstrated higher psychological distress and a higher rate of emotional eating than those with no lifestyle changes. The relationship between lifestyle changes, psychological distress, and emotional eating was moderated by gender and COVID-19-related stressors.

Last, in a study investigating associations among economic status deterioration, mental health, emotional eating, and gender during the COVID-19 pandemic in 1807 participants [43], women were found to report higher mental health impairment (including greater prevalence of emotional eating) than men. However, men whose economic status significantly deteriorated reported high mental health impairment (emotional eating and adjustment difficulties) similar to women in the same situation.

Emotional eating during periods of collective crisis may be understood as a maladaptive coping mechanism linked to disruptions in emotion regulation processes. Heightened levels of stress, anxiety, and trauma-related symptoms may increase vulnerability to using food as a means of regulating negative affects. In this context, eating may serve to temporarily reduce emotional distress, provide comfort, or restore a subjective sense of control amid uncertainty and loss of structure. At the same time, disruptions in daily routines, reduced social support, and increased exposure to stressors may further reinforce reliance on eating-related behaviors as a readily available coping strategy.

In summary, the COVID-19 pandemic in Israel was characterized by a sharp and sustained increase in ED-related treatment-seeking among adolescents and adults across ambulatory and inpatient settings. Greater ED-related symptom severity and higher rates of psychiatric comorbidity were observed compared to the pre-pandemic period and were closely linked to pandemic-related anxiety and post-traumatic stress symptoms. High rates of emotional eating were of particular relevance during the time of lockdown, social distancing, and high emotional distress [33]. The existing medical and mental health infrastructure was ill-prepared for the sudden escalation in demand, and the waiting times for treatment increased substantially. In response, several rapid and partial service adaptations were implemented, including telemedicine-based group interventions for parents of adolescents with EDs and intensified ED-focused care delivered within general pediatric departments for hospitalized patients.

The pandemic necessitated a rapid shift toward telemedicine, a modality that had been minimally used in ED care in Israel prior to COVID-19. Although some populations, particularly adolescents, adapted relatively quickly to remote treatment [31], telehealth posed significant challenges for adult populations [31], most notably for Jewish ultra-Orthodox communities facing technological, cultural, and contextual barriers to engagement in telehealth [44].

Taken together, the findings from the COVID-19 period indicate a consistent increase in eating-related disturbances, particularly among adolescents and young adults, as well as greater clinical severity and service demands. At the same time, variability across studies and the predominance of observational data highlight important gaps in understanding long-term trajectories and differential vulnerability across populations.

These findings should be interpreted in light of considerable methodological variability across studies, including differences in sample characteristics, measurement tools, and study designs. This variability highlights the need for caution in drawing definitive conclusions while pointing to a consistent overall trend toward increased vulnerability to eating-related disturbances during periods of prolonged collective stress.

### 3.3. Eating Disturbances in Israel During the October 2023 Attack and War

The Swords of Iron War, which erupted following an unprecedented Hamas attack on southern Israel on 7 October 2023, together with the escalation involving Hezbollah and Iran, plunged Israel into a profound existential, psychological, and social crisis. This crisis was related, at least in part, to the fact that Israel was largely unprepared for the scale and nature of the attack and the prolonged duration of the war, with ceasefires achieved only intermittently. Secondly, Israel faced a persistent reality of uncertainty, ambiguity, and unpredictability, in which soldiers continued to be killed or wounded and civilians were repeatedly exposed to displacement, injury, and death. These conditions were further compounded by intense internal societal debate and by international controversy surrounding the war and its objectives.

Studies conducted in the first months following the 7 October 2023 attack pointed to a sharp and alarming increase in mental health morbidity across all population groups in Israel. Compared to the preceding 12 months, the proportion of adult Israeli citizens reporting probable PTSD symptoms nearly doubled (from 16.2% to 29.8%). Similarly, the rates of depressive symptoms increased from 31.3% to 44.8%, and of anxiety symptoms from 24.9% to 42.7% [13]. As the war continued, further increases were observed in PTSD, depression, anxiety, sleep disturbances, fatigue, and concentration difficulties, particularly among women, young adults, residents of conflict zones, and individuals experiencing traumatic loss, displacement, or economic hardship [12,16,45,46]. Additional emerging work has highlighted the high rate of moral injury, secondary trauma, and compassion fatigue among mental health professionals treating war-affected populations [47,48,49].

This ongoing reality of insecurity, traumatic stress, loss, social disruption, and sustained fear, combined with severe disturbances to daily routines, created fertile ground for both the exacerbation of pre-existing psychiatric conditions and the emergence of maladaptive coping behaviors, including unhealthy eating patterns and other eating-related disturbances [24,33,34,36]. Although systematic epidemiological data on EDs during the October war are not yet available, clinical impressions reported by ED treatment professionals across Israel suggest a marked increase in ED-related referrals, further burdening the already overstretched specialized ED services [36]. This pattern parallels the findings of general mental health services. For example, a retrospective cohort analysis using electronic health records from Israel’s largest healthcare organization documented a 23.2% increase in requests for mental health outpatient services between October and December 2023, compared with the same period in 2022 [16]. Initial empirical studies conducted during the October 2023 war reported substantial changes in health-related behaviors and lifestyle patterns. A large community-based survey of residents of a town near the Gaza border found that over one-third of the respondents reported weight changes since the onset of the war, and 63% reported significant deterioration in sleep quality [50]. Moreover, a considerable proportion of participants reported increased emotional eating or marked disruption of regular eating routines, patterns likely to elevate the risk for the development or exacerbation of EDs, particularly under conditions of prolonged stress and ongoing crisis [45,46,50].

Consistent findings emerged from a study of 575 students at Ashkelon Academic College, located in close proximity to the Gaza front lines. This study demonstrated that emotional eating as a means of self-soothing was particularly prevalent during the war, especially among women, younger students, and individuals reporting body dissatisfaction or a higher body mass index (BMI) [12,51]. Furthermore, students who reported higher perceived stress levels were significantly more likely to rely on food as a coping strategy [12,51].

Recent emerging research conducted during the ongoing war sought to clarify the psychological mechanisms linking prolonged trauma exposure with eating-related disturbances. A network analysis examining responses to trauma during the current conflict revealed marked gender differences in the patterns of adjustment, sleep, and eating-related disturbances [45]. Women exhibited significantly higher levels of night eating, emotional eating, sleep problems, perceived stress, adjustment difficulties, and PTSD-related symptoms, whereas men reported higher levels of direct exposure to traumatic events. Moreover, in women, PTSD symptoms and adjustment difficulties occupied a central and highly interconnected position, with rigid links between PTSD and anxiety symptoms, suggesting a more systemic and self-reinforcing pattern of emotional dysregulation, affecting dysregulated eating. By contrast, in men, direct exposure to traumatic events played a more influential role.

Complementing these findings, another recent cross-sectional study (n = 426) examined the influence of the interplay among childhood trauma, war exposure, and coping on the occurrence and severity of emotional eating [46]. The results indicated that childhood trauma, particularly emotional abuse, was significantly associated with both PTSD symptoms and emotional eating during the war. PTSD symptoms fully mediated the relationship between childhood trauma and emotional eating, underscoring their central role as a psychological mechanism linking early adversity to maladaptive coping in adulthood. Although war exposure significantly predicted PTSD severity, it did not moderate the association between PTSD and emotional eating, suggesting that once PTSD symptoms are activated, emotional eating can be considered a relatively stable trauma-related coping response. Consistent with prior findings, women reported higher levels of PTSD symptoms and emotional eating than men [46].

Together, the findings from the October 2023 war and similar findings from the COVID-19 pandemic [42] position emotional eating as a salient, gender-sensitive manifestation of trauma-related distress during collective crises, shaped by both current exposure to war conditions and earlier developmental vulnerabilities. As emotional eating is related to poor emotional regulation skills [42], it can be considered, in such times, a maladaptive stress regulator in the face of heightened overall distress.

Recent research has further emphasized that among populations directly exposed to military attacks, particularly those experiencing physical injury, bereavement, or displacement, the risk of developing PTSD, depression, and anxiety is substantially elevated [17], with heightened vulnerability observed among women, young adults, and individuals with a history of psychological difficulties [17]. Although no direct association between PTSD and EDs was established in this study, a robust body of literature has linked trauma-related symptoms to DE behaviors [24,33,34,45,46,52].

A distinct and clinically salient challenge emerged during the October 2023 war among individuals with pre-existing EDs. Large numbers of civilians from different regions in Israel were forcibly displaced from their damaged or destroyed homes and relocated to hotels or other temporary accommodations. Individuals with EDs were suddenly required to eat in unfamiliar, crowded dining environments, often alongside strangers. These conditions frequently led to symptomatic relapses or exacerbation of ED symptoms, accompanied by intense feelings of guilt and shame, as these individuals were preoccupied with their own eating-related distress while witnessing the war-related profound loss and suffering of most other people in their immediate surroundings. This situation was further complicated, as many of these patients’ therapists and dietitians were themselves displaced, recruited to army service, or otherwise unavailable, leaving them without consistent professional support at a time of heightened vulnerability [53]. Nonetheless, the parents of some adolescents with EDs who were already well engaged in treatment prior to the war were able to assume a supportive role in maintaining structured eating patterns when formal treatment was abruptly interrupted because of the war [54,55].

Within the context of the still ongoing October 2023 war and its continued escalation, it is essential to interpret current findings on eating-related disturbances with caution. At the time of writing, Israeli civilians remain exposed to sustained and recurrent threats, including ongoing missile attacks from Iran and Lebanon, and prolonged disruptions to daily life. Such conditions reflect an active and evolving traumatic context rather than a completed event. Accordingly, the observed increases in emotional eating, disrupted eating patterns, and ED-related symptomatology may represent early or acute stress responses. The longer-term trajectory of these disturbances, including their potential consolidation into chronic EDs or their interaction with enduring PTSD symptoms, cannot yet be fully determined. In line with these results, international research has demonstrated a strong relationship between trauma exposure, PTSD symptoms, and disordered eating behaviors [19,20,21].

Findings emerging from the ongoing war suggest a marked escalation in psychological distress accompanied by increased vulnerability to eating-related disturbances, particularly in populations exposed to prolonged threat and disruption. However, the current evidence remains limited and largely based on early or cross-sectional data, underscoring the need for longitudinal research to better understand the persistence, severity, and broader impact of these disturbances.

Taken together, the available findings suggest a convergence between trauma-related distress and eating-related pathology, although current evidence remains largely cross-sectional and preliminary. These considerations highlight the need for longitudinal research to better understand causal pathways and long-term outcomes to capture the delayed and cumulative eating-related effects of prolonged exposure to war conditions.

### 3.4. Treatment of Eating-Related Disturbances During the COVID-19 Pandemic and the October 2023 War

The treatment of EDs during the COVID-19 pandemic and the October 2023 war in Israel has not yet been systematically evaluated. We found only a few relevant studies by reviewing the published literature in English and Hebrew. In addition, insights were based on clinical observations and reports from Israeli ED specialists regarding their experiences during these crises. Altogether, the available evidence and clinical observations suggest that, due to the specific circumstances during the crises, treatment providers and services tended to use several modalities that were relatively uncommon in Israeli ED services before 2020.

### 3.5. Telehealth

During the COVID-19 lockdowns, routine face-to-face treatment was largely suspended due to infection risks, and both ambulatory and inpatient ED services shifted toward telehealth-based care, a modality that was relatively novel for ED treatment in Israel at the time [31]. Although all patients were offered the option to participate in online video sessions, utilization of telehealth services was partial rather than universal. For example, one study found that only around 35% of all therapy sessions in 2020, and 20% in 2021 were conducted remotely (see Table 2 [31]). A survey examining attitudes toward remote treatment among patients with AN revealed a clear generational divide: Adolescents generally adapted more positively to telehealth, whereas adult patients reported greater difficulty adjusting and lower satisfaction with online care [31]. In another study of adult patients with EDs who had to abruptly transition from face-to-face to telehealth intervention [36], around 70% stated that they would not choose to continue online therapy given the option. Longer duration of treatment, stronger therapeutic alliance, and higher COVID-19 anxiety were linked with more positive views toward the transition. Within the Jewish ultra-Orthodox population, the implementation of home-based virtual hospitalization models during the pandemic posed unique cultural and practical challenges, including limited access to technology, crowded living conditions, reduced privacy, and religiously informed reservations regarding online treatment [44].

Despite the difficulties associated with telemedicine use in patients with EDs, clinical observations and preliminary reports suggest that, in select cases, remote care yields unexpected therapeutic benefits, facilitating emotional openness among patients with AN who struggle with intimacy and being seen by their treatment providers amid increasing weight [44]. Some patients repeatedly found the option to turn off the cameras during sessions particularly relieving, especially during periods of weight restoration when concerns about being visibly observed intensified [44]. From a different angle, telehealth enabled intensive and ongoing parental involvement in treatment during the pandemic, and, when required and agreed upon, actual assistance from the treatment team to the parents during meal supervision [31,44,56].

One study assessed the feasibility and effectiveness of home-based telehealth supervision and treatment during the COVID-19 pandemic. Jewish ultra-Orthodox female adolescents and young adults hospitalized in a specialized ED department because of AN and their treatment providers had to be confined to their homes because of the pandemic, and the department was closed for about a month. A home-based telehealth intervention was rapidly developed in line with the department’s treatment principles during regular times [44]. The patients were weighed in clinics near their homes once weekly. Regular supervision sessions, including psychiatrists, clinical dietitians, and nurses, were held once weekly to check on the patients’ eating-related condition (including changes in weight) and their overall emotional well-being (including psycho-pharmacological consultation). Daily supervisions were held with the patients by the department’s nurses and with the families by the clinical dietitians. Telehealth psychotherapy was provided twice weekly, and parental consultation once weekly by the patients’ psychotherapists. Telehealth schooling was also conducted regularly. When the department reopened, no significant deterioration was observed among the 15 patients included in this program. Most patients and parents regarded the program as useful and were satisfied with it, despite the inherent problems associated with its implementation in Jewish ultra-Orthodox families who often live in small, overcrowded apartments with many children.

Regarding the October 2023 war, a large-scale retrospective cohort study examining 7.19 million healthcare interactions from an Israeli health organization serving approximately one-third of the national population compared telehealth utilization across three time periods: the first month of the war, the month preceding it, and the corresponding period of the previous year [57]. The use of telemedicine increased significantly during the war, particularly in primary conflict zones. Remote mental health consultations tripled, and nutrition services showed the highest rates of telemedicine adoption. These trends paralleled the broader rise in mental health morbidity and were especially notable for eating-related disturbances during the October 2023 war. Marked increases were also observed in telehealth utilization within family medicine, pediatric, and gynecology services, reflecting a system-wide shift toward remote care during crisis conditions [57].

To promote treatment-seeking behavior during the war, a brief 2.5 min video intervention was developed, featuring the personal testimony of a survivor of a terrorist attack describing his traumatic experiences and subsequent mental health struggles. This intervention was administered to individuals residing in active conflict zones and was compared with a psycho-educational control condition. The video elicited a significant immediate increase in the intention to seek mental health treatment relative to the control group. However, this effect was not sustained at the 30-day follow-up, suggesting that although brief narrative-based interventions may enhance short-term motivation, additional strategies are required to support long-term engagement with care [58].

### 3.6. Ambulatory and Day Treatments

Ambulatory day treatment programs have been expanded during the October 2023 war across Israel for both adolescents and adults with EDs in response to a substantial surge in referrals for ED treatment, prolonged waiting times, and the urgent need to prevent unnecessary inpatient hospitalizations while providing comprehensive, intensive care. These programs offer a structured and flexible continuum of care, enabling either an earlier step-down transition from full inpatient hospitalization or serving as an effective alternative to hospitalization during clinical deterioration. Importantly, these specialized services have been formally approved and reimbursed by Israel’s health maintenance organizations in collaboration with the Ministry of Health, reflecting the system’s recognition of their clinical value and role in alleviating pressure on inpatient ED services during times of crisis.

### 3.7. Alternatives to Inpatient Eating Disorder Treatment

In recent years, particularly during the COVID-19 pandemic and the October 2023 war, several alternatives to inpatient treatment for EDs have been developed and implemented. First, community-based intensive treatment centers (often referred to as balancing or adjustment centers) provide time-limited, 24-h therapeutic care for individuals with EDs for approximately 2–3 months, while allowing the patients to remain in their community. Admission to these centers requires prior medical stabilization; therefore, patients do not require acute inpatient hospitalization. Currently, approximately five such centers operate across Israel, each serving 10–15 patients. These programs are financially supported by national healthcare services, reflecting the growing recognition of their role as viable alternatives to inpatient care.

Second, home-based treatment services, which have long existed for various medical conditions, have expanded to include ED care primarily in the past two years. These services involve a multidisciplinary team, typically including general practitioners or pediatricians, psychiatrists, psychotherapists, and clinical dietitians, as well as structured home-based meal supervision, providing a wraparound, flexible intervention tailored to individual clinical needs. Despite their clinical potential, home-based ED services are only partially reimbursed by health insurance and remain largely privatized, limiting equitable access.

### 3.8. Targeted Short-Term Interventions

Several targeted short-term intervention models have been introduced and expanded. One approach involves telemedicine-based group interventions for parents only, grounded in the principles of family-based therapy (FBT). In this model, patients are weighed and medically monitored in community settings, while parents receive focused guidance and support to restore healthy eating behaviors.

One study [59] evaluated the outcomes of a structured waiting-list intervention for adolescents awaiting entry into specialized ED treatment during the COVID-19 pandemic. A retrospective analysis was conducted on 56 adolescents who participated in this intervention between July 2020 and March 2022. The intervention included weekly nutritional monitoring and virtual psychoeducational support groups for the parents. The findings indicated that most patients showed no clinical deterioration and moderate improvements in symptom severity and BMI. During the waiting period, weight and BMI increased, particularly among those whose parents attended more group sessions than others. Additionally, 23% of patients were redirected to lower-intensity care based on clinical assessment.

Another approach employs intensive, multidisciplinary, short-term, ED-focused interventions within general pediatric departments, with the explicit goal of preventing admission to specialized ED inpatient units. These teams typically include pediatricians, psychiatrists, psychotherapists, and clinical dietitians who work collaboratively to stabilize patients during the acute phase.

Owing to the ongoing shortage of trained mental health professionals, compounded by the protracted nature of the October 2023 war, a current priority within Israeli mental health services has been the training of less experienced practitioners, including graduate-level students in psychology, social work, expressive therapies, and nursing, to deliver brief, structured, evidence-informed interventions to individuals presenting with low to moderate levels of post-traumatic distress [16]. Within the field of EDs, such short-term telemedicine-based counseling may be delivered by appropriately trained mental health professionals and dietitians with some expertise in FBT, interpersonal psychotherapy (IPT), or cognitive-behavioral therapy (CBT), targeting individuals with less severe ED symptomatology and their parents. Further empirical research is required to evaluate the feasibility, effectiveness, and ethical implications of these approaches. However, given the medical and psychiatric complexities of EDs, task-shifting approaches must be implemented cautiously. Such models require clearly defined competency thresholds, structured training, and ongoing supervision by specialized clinicians to ensure patient safety and treatment quality. These approaches should complement, rather than replace, specialist care, particularly for moderate-to-severe cases.

In addition, Israeli health services have implemented in-depth training programs in EDs for mental health clinicians and multidisciplinary community-based teams to expand workforce capacity and enhance the quality of ED care during prolonged national crises. Notably, the available data regarding the socioeconomic and demographic characteristics of individuals accessing ED services in Israel during periods of collective crisis remains limited and inconsistent. In particular, there is a lack of systematic information on socioeconomic status, educational background, and geographic disparities (e.g., urban versus peripheral regions) in access to care in this country. This gap is especially important given the likelihood that structural and socioeconomic factors may influence both vulnerability to DE and access to specialized services. Therefore, future research is needed to better characterize these dimensions and inform equitable service planning and policy development.

## 4. Limitations

Several limitations should be acknowledged. First, the available literature is characterized by a predominance of studies focusing on AN, reflecting the current state of research in the Israeli context, particularly during periods of collective crisis. Other eating disorders, such as BN, binge ED, and avoidant/restrictive food intake disorder (ARFID), remain comparatively underrepresented and warrant further investigation.

Second, most studies lack comparison groups, which limits the ability to isolate the specific contribution of collective crises to the observed changes. At the same time, it should be noted that in the Israeli context, the population has been broadly exposed to prolonged and ongoing collective stress, making the identification of unaffected comparison groups inherently challenging.

Third, the literature may be subject to publication bias, with a tendency to report increased pathology during crisis periods.

Fourth, several of the studies included in this review are co-authored by the present authors. This situation reflects, in part, the central role of local Israeli experts in advancing ED research in times of crisis; nevertheless, efforts have been made to synthesize the findings in a balanced and critical manner, and all relevant available studies have been considered.

Fifth, this narrative review is limited by the lack of systematic assessment of the impact of the crises on eating-related disturbances and their treatment. Nevertheless, Israel offers a unique context in which two major collective crises have occurred in close succession.

Sixth, the current paper was written in close temporal proximity to the ongoing war and its evolving consequences. As emphasized in previous research on large-scale trauma exposures, the full psychiatric impact of such events often unfolds gradually over extended periods of time [60].

Last, given that most currently available data regarding the psychological impact of the ongoing war is cross-sectional or based on early clinical observations, causal conclusions cannot yet be drawn. Longitudinal studies are needed to determine the long-term impact of prolonged war-related stress on EDs, DE, and broader eating-related disturbances.

## 5. Conclusions

The present review offers several unique contributions to the existing literature. First, it integrates findings from two distinct yet consecutive large-scale crises within a single national context, allowing for a cumulative and comparative perspective of their impact on eating-related pathology. Second, it combines empirical evidence with clinical insights to capture both documented trends and emerging patterns not yet fully represented in the literature. Finally, by situating EDs within a broader socio-cultural and trauma-informed framework, this review highlights the importance of conceptualizing eating-related disturbances as part of the wider mental health response to prolonged crises.

Recent collective crises in Israel, most notably the COVID-19 pandemic and the October 2023 war, have profoundly shaped the mental health landscape and increased vulnerability to DE and EDs. Periods of sustained stress, uncertainty, and social disruption are associated with increased prevalence, severity, and clinical complexity of eating-related disturbances, ranging from subclinical DE to full-threshold EDs, alongside disruption of healthy eating patterns.

Individuals with pre-existing EDs were particularly vulnerable, experiencing symptom exacerbation, relapses, and barriers to care, while new cases emerged among those without prior diagnoses, reflecting maladaptive coping in the context of prolonged stress and reduced psychological resources. These trends occurred within an already strained mental health system, highlighting gaps in capacity, accessibility, and preparedness.

At the same time, the Israeli experience demonstrates the adaptive potential of mental health systems during a crisis. The expansion of telemedicine, and the development of ambulatory and day programs, alternatives to inpatient care, and targeted short-term interventions illustrate how necessity can drive more flexible and trauma-informed models of care, although their effectiveness has yet to be systematically evaluated.

Given the growing evidence of increased prevalence across the ED spectrum during collective crises, EDs should be prioritized by policymakers. Early identification, timely referral, and accessible, intensive treatment are essential to prevent deterioration and chronicity.

At present, it remains too early to determine the long-term trajectory of trauma-related psychopathology in the Israeli population or the enduring interaction between PTSD symptoms and eating-related disturbances. In particular, potential intergenerational implications of collective crises, an issue of particular relevance in the Israeli context given the historical layering of collective traumatic experiences [61], should be adequately assessed. Considering the growing body of evidence linking trauma exposure, PTSD symptomatology, and DE, it is plausible that the full scope and severity of eating-related disturbances associated with the current crises may only emerge in longitudinal studies conducted in the coming years.

From a clinical and policy perspective, the findings underscore the need for proactive and coordinated responses to eating-related disturbances during periods of collective crisis. Doing so would include the implementation of early identification and screening strategies, particularly among high-risk populations such as adolescents and individuals with pre-existing EDs living in at-risk regions. Health systems should prioritize expanding accessible, timely services, including telehealth and community-based interventions, while reducing waiting times and barriers to care. In addition, there is a need to develop scalable, trauma-informed treatment models and to strengthen workforce training and system preparedness to better address surges in demand during large-scale emergencies.

In conclusion, while trauma-related psychopathology has received considerable clinical and policy attention during such periods, EDs and DE have received comparatively less attention in the Israeli context, despite accumulating evidence of their increase during periods of collective crisis.

Addressing EDs in the context of collective trauma requires a shift from reactive to proactive planning within mental health systems. Future preparedness should prioritize scalable, multidisciplinary care models, workforce training, and continuity of treatment for vulnerable populations, with relevance extending beyond Israel to other settings facing prolonged collective crises.

## Figures and Tables

**Table 1:** Rate of new visits because of EDs per 1000 Maccabi Health Service members per year [32].

Year	Rate per 1000 Members Total	Rate per 1000 Members Females	Rate per 1000 Members Males
2017	221.1	270.9	129.7
2018	207.5	252.1	125.6
2019	202.7	267.3	107.6
2020	249.6	309.1	134.6
2021	284.2	351.4	126.3

Note: Rates are presented per 1000 members in the relevant age group, not the total population, which accounts for the relatively high values.

**Table 2:** Number of sessions and patients with anorexia nervosa treated by the ambulatory eating disorders (ED) services at the Sheba Medical Center, Tel Hashomer, Israel, between 2019–2021 [31].

Ambulatory ED Services			2019	2020	2021
Adults	Patients	Total	629	618	822
		New	438	404	605
	Sessions	Total	9896	10,841	15,182
		Telemedicine	—	4112	3556
Adolescents	Patients	Total	302	287	398
		New	186	155	214
	Sessions	Total	5521	7539	8600
		Telemedicine	—	2519	1539

Note: Telemedicine refers to remotely delivered treatment sessions.

## Data Availability

No new data were created or analyzed in this study.

## References

[1] Sher L. (2020). The impact of the COVID-19 pandemic on suicide rates. QJM An. Int. J. Med..

[2] Farooq S., Tunmore J., Ali M.W., Ayub M. (2021). Suicide, self-harm and suicidal ideation during COVID-19: A systematic review. Psychiatry Res..

[3] Bilu Y., Flaks-Manov N., Bivas-Benita M., Akiva P., Kalkstein N., Yehezkelli Y., Mizrahi-Reuveni M., Ekka-Zohar A., David S.S., Lerner U. (2023). Data-driven assessment of adolescents’ mental health during the COVID-19 pandemic. J. Am. Acad. Child Adolesc. Psychiatry.

[4] Yan Y., Hou J., Li Q., Yu N.X. (2023). Suicide before and during the COVID-19 pandemic: A systematic review with meta-analysis. Int. J. Environ. Res. Public Health.

[5] Termorshuizen J.D., Watson H.J., Thornton L.M., Borg S., Flatt R.E., MacDermod C.M., Harper L.E., van Furth E.F., Peat C.M., Bulik C.M. (2020). Early impact of COVID-19 on individuals with self-reported eating disorders: A survey of ~1000 individuals in the United States and the Netherlands. Int. J. Eat. Disord..

[6] Rodgers R.F., Lombardo C., Cerolini S., Franko D.L., Omori M., Fuller-Tyszkiewicz M., Linardon J., Courtet P., Guillaume S. (2020). The impact of the COVID-19 pandemic on eating disorder risk and symptoms. Int. J. Eat. Disord..

[7] Branley-Bell D., Talbot C.V. (2020). Exploring the impact of the COVID-19 pandemic and the UK lockdown on individuals with experience of eating disorders. J. Eat. Disord..

[8] Clark Bryan D., Macdonald P., Ambwani S., Cardi V., Rowlands K., Willmott D., Treasure J. (2020). Exploring the ways in which COVID-19 and lockdown have affected the lives of adult patients with anorexia nervosa and their carers. Eur. Eat. Disord. Rev..

[9] Fernández-Aranda F., Casas M., Claes L., Bryan D.C., Favaro A., Granero E., Gudiol C., Jiménez-Murcia S., Karwautz A., Le Grange D. (2020). COVID-19 and implications for eating disorders. Eur. Eat. Disord. Rev..

[10] Schlegl S., Meule A., Favreau M., Voderholzer U. (2020). Bulimia nervosa in times of the COVID-19 pandemic—Results from an online survey of former inpatients. Eur. Eat. Disord. Rev..

[11] Baenas I., Etxandi M., Munguía L., Granero R., Mestre-Bach G., Sánchez I., Ortega E., Andreu A., Moize V.L., Fernández-Real J.M. (2021). Impact of COVID-19 lockdown in eating disorders: A multicenter collaborative international study. Nutrients.

[12] Dopelt K., Houminer-Klepar N. (2024). War-related stress among Israeli college students following the 7 October 2023 terror attack in Israel. Eur. J. Investig. Health Psychol. Educ..

[13] Levi-Belz Y., Groweiss Y., Blank C., Neria Y. (2024). PTSD, depression, and anxiety after the October 7, 2023, attack in Israel: A nationwide prospective study. eClinicalMedicine.

[14] Aida T. (2020). Revisiting suicide rate during wartime: Evidence from the Sri Lankan civil war. PLoS ONE.

[15] Levany S., Shahar G., Greenberg D. (2023). Calling for an immediate release of captive children in Gaza. Lancet.

[16] Neria Y., Markowitz J.C., Amsalem D., Levi-Belz Y., Roe D., Lurie I., Bitan D.T., Wainberg M.L., Mendlovic S. (2025). Israeli mental health in the aftermath of the October 7 terrorist attack: Risks, challenges, and recommendations. Isr. J. Health Policy Res..

[17] Levi-Belz Y., Blank C., Groweiss Y., Neria Y. (2024). The impact of PTSD symptoms on suicide ideation in times of terror and war: A nationwide prospective study on the moderating role of loneliness. Psychiatry Res..

[18] Fox V., Dalman C., Dal H., Hollander A.C., Kirkbride J.B., Pitman A. (2021). Suicide risk in people with post-traumatic stress disorder: A cohort study of 3.1 million people in Sweden. J. Affect. Disord..

[19] Brewerton T.D. (2007). Eating disorders, trauma, and comorbidity: Focus on PTSD. Eat. Disord..

[20] Mitchell K.S., Scioli E.R., Galovski T., Belfer P.L., Cooper Z. (2025). Posttraumatic stress disorder and eating disorders: Maintaining mechanisms and treatment targets. Trauma, PTSD and Eating Disorders.

[21] Molendijk M.L., Hoek H.W., Brewerton T.D., Elzinga B.M. (2017). Childhood maltreatment and eating disorder pathology: A systematic review and dose-response meta-analysis. Psychol. Med..

[22] Van Strien T., Roelofs K., de Weerth C. (2013). Cortisol reactivity and distress-induced emotional eating. Psychoneuroendocrinology.

[23] Evers C., Dingemans A., Junghans A.F., Boevé A. (2018). Feeling bad or feeling good: Does emotion affect your consumption of food? A meta-analysis of the experimental evidence. Neurosci. Biobehav. Rev..

[24] Pat-Horenczyk R., Peled O., Miron T., Brom D., Villa Y., Chemtob C.M. (2007). Risk-taking behaviors among Israeli adolescents exposed to recurrent terrorism: Provoking danger under continuous threat?. Am. J. Psychiatry.

[25] Bachar E., Canetti L., Berry E.M. (2005). Lack of long-lasting consequences of starvation on eating pathology in Jewish Holocaust survivors of Nazi concentration camps. J. Abnorm. Psychol..

[26] Latzer Y., Azaiza F., Tzischinsky O. (2009). Eating attitudes and dieting behavior among religious subgroups of Israeli-Arab adolescent females. J. Relig. Health.

[27] Latzer Y., Weinberger-Litman S.L., Gerson B., Rosch A., Mischel R., Hinden T., Kilstein J., Silver J. (2015). Negative religious coping predicts disordered eating pathology among Orthodox Jewish adolescent girls. J. Relig. Health.

[28] Latzer Y., Stein D., Witztum E. (2019). Treating ultra-orthodox adolescents with eating disorders in Israel: Culturally-sensitive interventions, difficulties, and dilemmas. J. Clin. Psychol..

[29] Latzer Y., Stein D. (2024). Overview of disordered eating and eating disorders in Israel: Prevalence and treatment. Eating Disorders: An International Comprehensive View.

[30] Latzer Y., Witztum E., Stein D. (2008). Eating disorders and disordered eating in Israel: An updated review. Eur. Eat. Disord. Rev. Prof. J. Eat. Disord. Assoc..

[31] Serur Y., Dikstein H., Shilton T., Gothelf D., Latzer Y., Lewis Y., Enoch-Levy A., Pessach I., Gur E., Stein D. (2022). The emotional-behavioral state of Israeli adolescent and young adult females with anorexia nervosa during the COVID-19 pandemic. J. Eat. Disord..

[32] Peleg-Gabay M., Levy S. (2022). The outline of treatment services for eating disorders in Israel. The Israel.

[33] Alon-Tirosh M., Hadar-Shoval D., Asraf K., Tannous-Haddad L., Tzischinsky O. (2021). The association between lifestyle changes and psychological distress during COVID-19 lockdown: The moderating role of COVID-related stressors. Int. J. Environ. Res. Public Health.

[34] Efrati Y., Spada M.M. (2022). Self-perceived substance and behavioral addictions among Jewish Israeli adolescents during the COVID-19 pandemic. Addict. Behav. Rep..

[35] Goldberg L., Ziv A., Vardi Y., Hadas S., Zuabi T., Yeshareem L., Gur T., Steinling S., Scheuerman O., Levinsky Y. (2022). The effect of the COVID-19 pandemic on hospitalizations and disease characteristics of adolescents with anorexia nervosa. Eur. J. Pediatr..

[36] Lewis Y.D., Elran-Barak R., Grundman-Shem Tov R., Zubery E. (2021). The abrupt transition from face-to-face to online treatment for eating disorders: A pilot examination of patients’ perspectives during the COVID-19 lockdown. J. Eat. Disord..

[37] Elran-Barak R., Segel-Karpas D., Epstlein R. (2023). Health Behaviors during the Early COVID-19 Containment Phase and Their Impact on Psychological Health. Healthcare.

[38] Nudelman G., Peleg S., Shiloh S. (2021). The association between healthy lifestyle behaviors and coronavirus protective behaviors. Int. J. Behav. Med..

[39] Ammar A., Brach M., Trabelsi K., Chtourou H., Boukhris O., Masmoudi L., Bouaziz B., Bentlage E., How D., Ahmed M. (2020). Effects of COVID-19 home confinement on eating behavior and physical activity: Results of the ECLB-COVID-19 international online survey. Nutrients.

[40] Olfert M.D., Wattick R.A., Saurborn E.G., Hagedorn R.L. (2022). Impact of COVID-19 on college student diet quality and physical activity. Nutr. Health.

[41] Yehudai M., Clark M., Azzopardi A., Romem Porat S.L., Dagan A., Reznik A., Isralowitz R. (2023). COVID-19 fear impact on Israeli and Maltese female “help” profession students. Int. J. Environ. Res. Public Health.

[42] Hadar-Shoval D., Alon-Tirosh M., Asraf K., Tannous-Haddad L., Tzischinsky O. (2022). Lifestyle changes, emotional eating, gender, and stress during COVID-19 lockdown. Nutrients.

[43] Hadar-Shoval D., Alon-Tirosh M., Asraf K., Tannous-Haddad L., Tzischinsky O. (2022). The association between men’s mental health during COVID-19 and deterioration in economic status. Am. J. Men’s Health.

[44] Latzer Y., Herman E., Ashkenazi R., Atias O., Laufer S., Biran Ovadia A., Oppenheim T., Shimoni M., Uziel M., Stein D. (2021). Virtual online home-based treatment during the COVID-19 pandemic for ultra-orthodox young women with eating disorders. Front. Psychiatry.

[45] Spivak-Lavi Z., Tzischinsky O., Latzer Y., Shklarski L. (2026). Exploring Gendered Responses to Trauma: Network Analysis of Adjustment, Sleeping, and Eating Disturbances During Ongoing Conflict. Psychol. Trauma Theory Res. Pract. Policy.

[46] Spivak-Lavi Z., Latzer Y., Tzischinsky O. (2025). When childhood trauma meets war: Emotional eating through the lens of PTSD. Rambam Maimonides Med. J..

[47] Latzer Y., Shklarski L., Spivak-Lavi Z., Hinich S. (2025). Psychotherapists in Traumatological Environments: Coping Through Collective Mission, Shared Trauma, and Intergenerational Wounds. Clin. Soc. Work J..

[48] Shklarski L., Latzer Y., Spivak-Lavi Z., Tuson C. (2026). Weathering the storm together: Therapists’ experiences treating war trauma survivors while managing their own concurrent trauma. Stress Health.

[49] Shklarski L., Latzer Y. (2025). Compassion fatigue among Israeli therapists after the October 7 attack: Challenges in treating bereaved and hostage families. Psychol. Trauma Theory Res. Pract. Policy.

[50] Shamir-Stein N., Feldblum I., Rotman E., Cohen S., Brand E., Kivity S., Saban M. (2024). Exploring the self-reported physical and psychological effects in a population exposed to a regional conflict. J. Community Health.

[51] Houminer Klepar N., Davidovitch N., Dopelt K. (2024). Emotional eating among college students in Israel: A study during times of war. Foods.

[52] Convertino A.D., Morland L.A., Blashill A.J. (2022). Trauma exposure and eating disorders: Results from a United States nationally representative sample. Int. J. Eat. Disord..

[53] Lewis Y.D., Zubery E. (2024). The October massacre and iron swords war as a trigger for the emergence of eating disorders-a case series. Harefuah.

[54] Danziger Y., Ram A., Mimouni M. (1994). Outcome of Israeli adolescents with anorexia nervosa whose ambulatory treatment was abruptly interrupted during the Gulf War. Acta Paedopsychiatr..

[55] Serur Y., Enoch-Levy A., Pessach I., Joffe-Milstein M., Gothelf D., Stein D. (2021). Treatment of eating disorders in adolescents during the COVID-19 pandemic: A case series. J. Eat. Disord..

[56] Schlissel A.C., Richmond T.K., Eliasziw M., Leonberg K., Skeer M.R. (2023). Anorexia nervosa and the COVID-19 pandemic among young people: A scoping review. J. Eat. Disord..

[57] Sberro-Cohen S., Ellen M.E. (2025). From Conflict to Care-Telemedicine Utilization During Wartime: A Retrospective Cohort Study. J. Med. Syst..

[58] Amsalem D., Haim-Nachum S., Lazarov A., Levi-Belz Y., Markowitz J.C., Bergman M., Rafaeli A.K., Brenner L.A., Nacasch N., Wainberg M. (2025). Brief video intervention to increase treatment-seeking among individuals living in a conflict zone: A randomized controlled trial. Psychiatry Res..

[59] Lavan O., Goldstein A., Bar Eyal A., Shir A., Hammer T., Label A., Valik N., Ratzon R., Tahar T., Fennig S. (2025). Waiting list intervention for adolescents with anorexia nervosa. Eat. Disord..

[60] Ayhan C.H., Tanhan F., Yağan F., Avcı Erdal N., Öztürk G., Bedir G., Aslangiri S. (2025). 12 years after Roboski: Prolonged grief and posttraumatic effects. Psychol. Trauma Theory Res. Pract. Policy.

[61] Bachar E. (1994). Aggression expression in grandchildren of Holocaust survivors—A comparative study. Isr. J. Psychiatry Relat. Sci..

